# Mechanical Properties of Natural Fiber Reinforced Foamed Concrete

**DOI:** 10.3390/ma13143060

**Published:** 2020-07-08

**Authors:** Joaquin F. Castillo-Lara, Emmanuel A. Flores-Johnson, Alex Valadez-Gonzalez, Pedro J. Herrera-Franco, Jose G. Carrillo, P. I. Gonzalez-Chi, Q. M. Li

**Affiliations:** 1 Unidad de Materiales, Centro de Investigación Científica de Yucatán, Calle 43, No. 130 Col. Chuburná de Hidalgo, Mérida, Yucatán 97205, Mexico; joaquin.castillo6@outlook.com (J.F.C.-L.); avaladez@cicy.mx (A.V.-G.); pherrera@cicy.mx (P.J.H.-F.); jgcb@cicy.mx (J.G.C.); ivan@cicy.mx (P.I.G.-C.); 2 CONACYT-Unidad de Materiales, Centro de Investigación Científica de Yucatán, Calle 43, No. 130 Col. Chuburná de Hidalgo, Mérida, Yucatán 97205, Mexico; 3 Department of Mechanical, Aerospace and Civil Engineering, School of Engineering, The University of Manchester, Manchester M13 9PL, UK; Qingming.Li@manchester.ac.uk

**Keywords:** foamed concrete, natural fiber, henequen fiber, alkaline treatment, polypropylene fiber, mechanical properties, air-void structure, fiber reinforced foamed concrete

## Abstract

The mechanical characterization of plain foamed concrete (PFC) and fiber-reinforced foamed concrete (FRFC) with a density of 700 kg/m^3^ was performed with compression and tension tests. FRFC was reinforced with the natural fiber henequen (untreated or alkaline-treated) at volume fractions of 0.5%, 1% and 1.5%. Polypropylene fiber reinforcement was also used as a reference. For all FRFCs, the inclusion of the fibers enhanced the compressive and tensile strengths and plastic behavior, which was attributed to the increase of specimen integrity. Under compressive loading, after the peak strength, there was no considerable loss in strength and a plateau-like regime was observed. Under tensile loading, the fibers significantly increased the tensile strength of the FRFCs and prevented a sudden failure of the specimens, which was in contrast to the brittle behavior of the PFC. The tensile behavior enhancement was higher when treated henequen fibers were used, which was attributed to the increase in the fiber–matrix bond produced by the alkaline treatment. The microscopic characterization showed that the inclusion of fibers did not modify the air-void size and its distribution. Higher energy absorption was observed for FRFCs when compared to the PFC, which was attributed to the enhanced toughness and ductility by the fibers. The results presented herein warrant further research of FRFC with natural henequen fibers for engineering applications.

## 1. Introduction

Foamed concrete, also known as cellular concrete, is a cementitious material, in which, air-voids are trapped in the mortar. With the correct dosage of foam, the density of the concrete can vary between 400 and 1600 kg/m^3^ [1]. Foamed concrete has good workability and is classified as a lightweight concrete. It can be used in filling applications, thermal insulation, acoustic insulation, fire resistance and impact energy absorption applications; however, foamed concrete is not usually used as a structural material due to its low compressive strength. Moreover, foamed concrete is not good in supporting tensile loads; it often cracks in the plastic state, during drying shrinkage and in the hardened state. The flexural and tensile strengths of foamed concrete range between 15 and 35% of its compressive strength [2]. It has been found that the use of polymer fibers as reinforcement in a concrete matrix produces shrinkage crack reduction and improves the mechanical properties, in particular, the tensile and flexural properties [3].

In foamed concrete, the inclusion of fibers with random orientation can improve load transfer, compressive strength, tensile strength and ductility by changing the typical brittle behavior to elastic–plastic behavior [2,4]. These improvements are very important because they could support the development of foamed concretes for structural applications [5]. Flores-Johnson and Li [6] studied the inclusion of polyvinyl alcohol fibers with a volume fraction of 3.3% into foamed concrete with a density of 1000 kg/m^3^. They found that this reinforcement led to increases in compressive and tensile strengths of 84.7% and 558%, respectively, when compared to the unreinforced foamed concrete. They also found that the inclusion of fibers changed the brittle failure mode of foamed concrete to elastic–plastic behavior. Jones and McCarthy [7] reported the mechanical behavior of foamed concrete with a density of 1400 kg/m^3^ reinforced with polypropylene (PP) fibers at volume fractions of 0.25% and 0.5%. The most marked increases of mechanical performance were observed at 0.5% volume fraction, i.e., the compressive and flexural strengths were 52% and 58% higher than the reference, respectively. Othuman Mydin and Soleimanzadeh [8] found that the flexural strength of foamed concrete with densities in the range of 600–1400 kg/m^3^ improved with the inclusion of PP fibers using volume fractions of up to 0.4%. They also reported that for a density of 800 kg/m^3^, an increase in the fiber volume fraction (from 0.2 to 0.4%) produced a coarser pore structure, in particular in the areas around the fibers. Mugahed Amran et al. [9] investigated the mechanical properties of foamed concrete with densities in the range of 1000–1900 kg/m^3^ reinforced with PP fibers at volume fractions in the range of 0.5–3%. They found that the inclusion of the fibers at volume fractions of 0.75 and 1% improved the compressive and flexural strengths of the foamed concrete.

The most common types of fibers to reinforce concrete are metallic [10] and synthetic fibers [4,11]; however, it is increasingly common to use natural fibers as reinforcement. The interest in materials reinforced with natural fibers is growing because it is possible to obtain materials with good mechanical properties, low density and low cost. In addition, natural fibers are biodegradable, renewable and promote sustainability [12,13,14,15], which is increasingly important currently. These fibers could replace synthetic fibers in certain applications, since synthetic fibers leave a greater environmental footprint. Several studies have been performed on cement-based materials reinforced with natural fibers including sisal, coir, jute and kraft pulp fibers, among others. These studies have found that there is an enhancement of flexural, impact, tensile and compressive strengths and toughness of the cement-based materials due to the fiber-reinforcement [16,17,18], which is consistent to the findings when polymer fibers are used as reinforcement. However, there are concerns about the use of natural fibers in cement-based matrices due to the long-term durability of the reinforced materials that may undergo reduction in strength and toughness [19]. Natural fibers are highly sensitive to the alkalinity of the cementitious matrix, which results in fiber degradation and the reduction of the flexibility and deformation capacity of the fibers due to the embrittlement associated with the mineralization of the fibers [19]. The low degradation resistance of lignin and hemicellulose in highly alkaline environments can cause a decrease in integrity and stability of the cell wall of natural fibers in cement-based materials [20]. The durability of natural fiber-reinforced cementitious materials can be improved by polymer coating [21], chemical treatment [22], hornification [23] or thermal treatment [24] of the fibers.

As aforementioned, several studies have been performed to assess the mechanical performance of natural fiber reinforced cement-based materials; however, most of these studies have been carried out on mortar and concrete. Foamed concrete reinforced with natural fibers has been scarcely studied. Mahzabin et al. [25] found that foamed concrete with a density of 1250 kg/m^3^ and 0.45% kenaf fiber volume fraction, exhibited an increase in compressive strength when compared to the unreinforced foamed concrete. Othuman Mydin et al. [26] reported the mechanical properties of foamed concrete with densities in the range of 800–1250 kg/m^3^ reinforced with coir fiber at volume fractions of 0.2% and 0.4%. They found that the fiber-reinforcement improved the tensile, compressive and flexural strengths and impact resistance of the foamed concrete. Liu et al. [27] found that by adding 0.75% of sisal fiber to foamed concrete, the shrinkage performance and the mechanical properties of the material were improved. Amarnath and Ramachandrudu [28] reported that the use of sisal fiber as reinforcement in foamed concrete with a density of 1200 kg/m^3^ improved the compressive and tensile strengths. Flores-Johnson et al. reported an improvement of the mechanical properties of foamed concrete with densities of 800 kg/m^3^ [29] and 900 kg/m^3^ [30] with the inclusion of henequen fibers.

The motivation for this work is the lack of knowledge on low density foamed concrete, reinforced by natural fibers. Low density foamed concrete can be used as a lightweight construction material to effectively reduce the risk of earthquake damage [30,31], and as an energy absorbing material for impact applications [32]. Moreover, research on the use of the natural fiber henequen (*Agave fourcroydes* Lem.) to reinforce cement-based materials, in particular, foamed concrete, is very limited when compared to the research performed on sisal (*Agave sisalana* Perrine) fiber. Henequen is the main cultivated species of *Agave* for fiber production in the Yucatan peninsula in Mexico, endures drought better than sisal [33] and can grow in semi-arid, rocky and nutritionally poor soils. To address these issues, the mechanical characterization of plain and fiber-reinforced foamed concrete developed by the pre-foaming method with a target dry density of 700 kg/m^3^ is presented and discussed. Henequen (untreated and treated) fibers were analyzed as the reinforcement at fiber volume fractions of 0.5%, 1% and 1.5%. For the treated henequen fibers, an alkaline chemical treatment with an aqueous sodium hydroxide (NaOH) solution was used. Polypropylene (PP) fiber reinforcement was also used as a reference; PP fiber is a readily available construction material, which is commonly used for the reinforcement of cement-based materials. Uniaxial compression and tension tests were performed, and the results were analyzed and discussed. A microscopic characterization on the fiber-reinforced foamed concretes, showing the overall air-void size and its distribution, is also presented.

The research significance of this work is to advance the understanding of the mechanical behavior of natural fiber reinforced foamed concrete. There is currently a knowledge gap regarding the mechanical behavior of low density foamed concrete reinforced with treated henequen fibers at different volume fractions. Compression and tension tests performed on foamed concrete with a density of 700 kg/m^3^ and fiber volume fractions in the range of 0.5–1.5% demonstrates that the henequen fiber can be used to reinforce cement-based materials. The mechanical testing shows that the treated henequen fibers could potentially replace synthetic fibers in sustainable construction applications since they provide similar benefits in terms of compression properties and produce improved tensile properties. The use of natural fibers, which are low-cost sustainable materials, is of great value currently, i.e., it is increasingly important to study and develop sustainable materials for applications in the construction industry.

## 2. Materials and Methods

### 2.1. Foamed Concrete and Fibers

**Figure 1:**
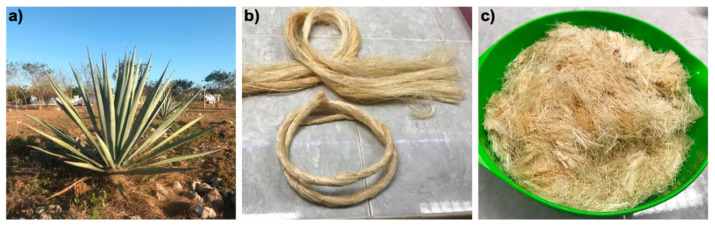
(**a**) Henequen plant; (**b**) henequen fibers and (**c**) henequen fibers chopped to a length of 19 mm.

The foamed concrete mix design utilized in this investigation is shown in Table 1, which is referred to as plain foamed concrete (PFC). The mixture was prepared using Portland cement, limestone aggregate, water and foam. The content (in mass) of the constituents is shown in Table 1. Portland cement CPC 30R EXTRA (Cemex, Merida, Mexico) with a minimum strength of 30 MPa at 28 days was used [34]. The chemical composition and granulometry of the cement and limestone aggregates can be found in [35] and [36], respectively. The aggregate was 25% in mass of coarse limestone aggregate (passing through the 1.19 mm sieve and retained on the 600 μm sieve) and 75% in mass of fine limestone aggregate (passing through the 600 μm sieve). For both PFC and fiber-reinforced foamed concrete (FRFC), a target dry density of 700 kg/m^3^ was used. For the FRFC, natural fibers extracted from the henequen plant (*Agave fourcroydes* Lem.) were used (Figure 1a). The henequen fibers were supplied by local producers in Baca, Yucatan (Desfibradora La Lupita, Baca, Mexico). The henequen fibers (Figure 1b) were chopped to a length of 19 mm (Figure 1c). Additionally, 19-mm long polypropylene (PP) fibers, Fibercon Microfibra (Dificonsa, Tlanepantla de Baz, Mexico) [37] were used as a reference; this is a readily available synthetic fiber, which is commonly used for the reinforcement of cement-based materials. The reported mechanical properties of both henequen and PP fibers are shown in Table 2 as a reference. All FRFC mixtures were prepared using the mix design shown in Table 1. Three different fiber volume fractions were used (0.5%, 1% and 1.5%). These fiber volume fractions were selected based on preliminary work [29] and previous investigations [27,28].

### 2.2. Alkaline Treatment of Henequen Fibers

For the treated fiber-reinforced mixtures, henequen fibers were treated with a sodium hydroxide (NaOH) aqueous solution at 2% (*w/v*; i.e., 2.04 g of NaOH per 100 mL of solution) for 1 h at 25 °C, using a mechanical stirrer at 550 rpm. Subsequently, the fibers were washed with water until all the sodium hydroxide was eliminated. Finally, the fibers were dried in an oven at 60 °C for 24 h [38].

### 2.3. Foamed Concrete Preparation

Foamed concrete with a target dry density of 700 kg/m^3^ was prepared using the pre-foaming method, which consisted of 3 stages: (1) preparation of the liquid cement mixture (mortar); (2) preparation of the foam and (3) mixing of the mortar and foam. The materials were mixed in a 60-litre mixer until a homogeneous mixture was obtained. The foam was prepared separately using a foam generator JFG200 (Propump Engineering, Crayford, UK) and EABASSOC foaming agent (EAB Associates, Altrincham, UK). To prepare the foam, a mixture of the foaming agent and water was prepared (31 mL of foaming agent per liter of water). This mixture was then poured into the foam generator container. The container was pressurized to 0.689 MPa (100 psi), which produced a foam density of 50 ± 2.5 kg/m^3^. Finally, the foam was poured into the mixer containing the mortar until a wet density of 800 kg/m^3^ was achieved. The FRFC mixtures were prepared following the same procedure as above with the fibers added to the mixture after stage 3. All the different foamed concrete mixtures prepared in this study are shown in Table 3.

### 2.4. Mechanical Characterization of Foamed Concrete

#### 2.4.1. Uniaxial Compression Test

The compression behavior of each mixture was evaluated in accordance with the BS EN 12390 standard using a Shimadzu AG-1 universal testing machine, equipped with a load cell of 100 kN, and a cross-head speed of 2.4 mm/min. It is noted that the compressive displacement of the specimens was measured using the crosshead displacement of the universal testing machine, and consequently, the accuracy in the measurement of elastic strains is limited. Cubical specimens with dimensions of 100 mm × 100 mm × 100 mm were used. The specimens were cured in molds for 1 day after casting; then, they were demolded and cured in a plastic bag for 24 days. After that, the specimens were removed from the bag and air cured for 3 days at a temperature of 25 °C. All samples were tested 28 days after casting.

#### 2.4.2. Uniaxial Tensile Test

Uniaxial tensile tests were performed using briquette specimens (dog-bone shaped specimens) with a length of 76 mm and a cross-section area of 25.4 mm × 25.4 mm in the middle section. All samples were tested 28 days after casting. A Shimadzu AG-1 universal testing machine with a 20-kN load cell was used. The load was applied at a constant displacement rate of 1 mm/min.

### 2.5. Microscopy

Scanning electron microscopy (SEM) analysis of foamed concrete samples was conducted on a low vacuum scanning electron microscope JEOL JSM 6360 LV (JEOL, Tokyo, Japan). Specimens with dimensions of 8 mm × 8 mm × 5 mm were cut from a foamed concrete briquette of each mixture. One specimen per mixture was obtained. Subsequently, the surface of the specimens was polished and cleaned with compressed air. Finally, the specimens were placed on a sample holder and coated with gold for better electron conductivity. Four SEM micrographs were taken for each specimen in order to study the cellular structure. To analyze the air-void size for each mixture, the total area *A_m_* of each air-void was measured using the image processing software ImageJ v. 1.60 (National Institutes of Health, Bethesda, MD, USA), and the equivalent diameter *d_e_* was obtained using the following equation [39]:

*d_e_* = (4 *A_m_*/π)^1/2^ (1)

The equivalent diameter provides an easy way to estimate the air-void diameter assuming that the void shape is spherical. Thirty-five measurements were made in each micrograph to obtain a total of 140 measurements for each mixture. The average diameter and histograms of the air-void diameter distribution were obtained from the measurements. The porosity of the air-voids *P* was calculated using the following equation [40]:

*P* = 1 − *(ρ_FOAM_*/*ρ_SOLID_*) (2)

where *ρ_FOAM_* is the dry density of foamed concrete. The average measured value of the solid density of the mortar without foam *ρ_SOLID_* was 1723 kg/m^3^.

## 3. Results and Discussion

### 3.1. Uniaxial Compression Test

**Figure 2:**
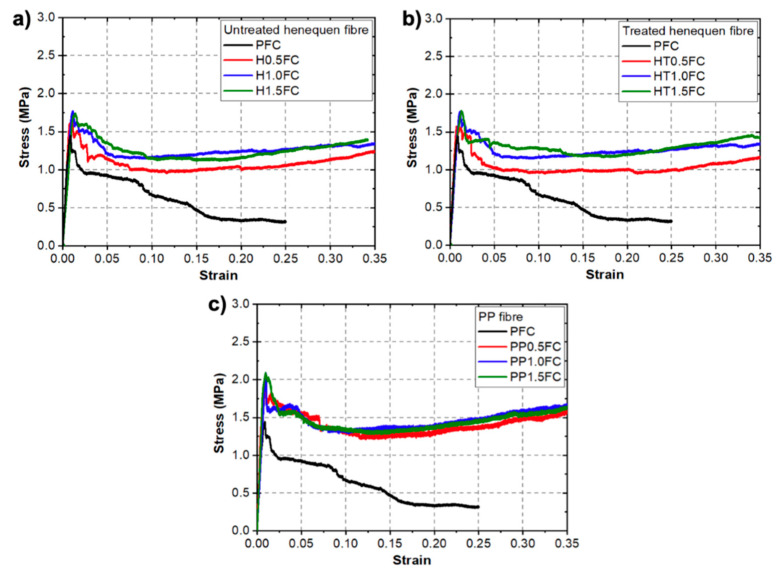
Typical uniaxial compression stress–strain curves for reinforced foamed concrete mixtures with different fiber-reinforcements and volume fractions. A stress–strain curve of the plain foamed concrete (PFC) is included in each figure for comparison purposes: (**a**) untreated henequen fiber; (**b**) treated henequen fiber and (**c**) PP fiber.

**Figure 3:**
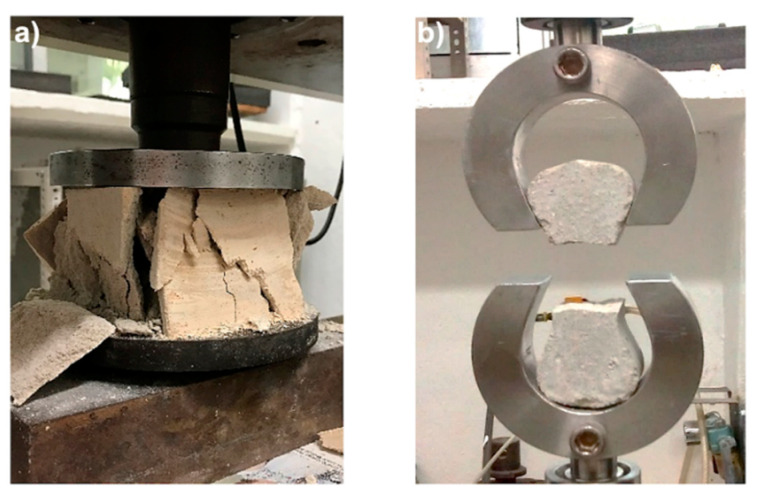
Plain foamed concrete (PFC) specimens: (**a**) after the compression test and (**b**) after the tension test.

Figure 2 shows typical stress–strain curves for the FRFCs under uniaxial compression. A stress–strain curve of the PFC is included in each figure for comparison purposes. For the PFC, it can be seen that after the material reached the maximum strength, it failed and gradually lost strength as the strain increased. At strains greater than 10%, the specimen started to lose its integrity and started to crumble (Figure 3a), which prevented the material from resisting the load at larger deformations. This type of failure is characteristic of foamed concrete without reinforcement [6]. A different behavior was observed for the FRFCs (Figure 2). For these materials, after the compressive peak strength was reached, the specimens were able to carry load at increasing strains. This behavior was attributed to the enhanced integrity of the specimens provided by the fibers, since the fiber reinforcement prevented cracks growing by forming connection bridges on the concrete.

**Figure 4:**
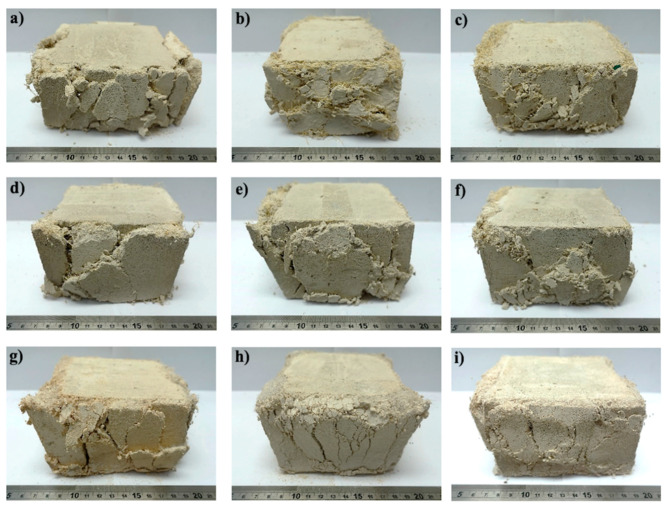
Deformation of the representative foamed concrete specimens at 0.35 compressive strain, reinforced with untreated henequen fiber: (**a**) H0.5FC; (**b**) H1.0FC and (**c**) H1.5FC; with treated henequen fiber: (**d**) HT0.5FC; (**e**) HT1.0FC and (**f**) HT1.5FC and with PP fiber: (**g**) PP0.5FC; (**h**) PP1.0FC and (**i**) PP1.5FC.

Figure 4 shows representative compressed specimens at a strain of 0.35. It is clear that the fibers prevented the separation of large concrete pieces after cracking, which is in contrast to the brittle behavior of PFC specimens (Figure 3a). The enhanced integrity of the specimens by the fiber-reinforcement is more evident for the specimens with volume fractions of 1% and 1.5%. It can also be seen in Figure 2 that, for the FRFCs, the load began to increase at a strain of 15–20%, which indicates that in some parts of the specimen, local densification strain was reached; this is a common characteristic of cellular solids [41]. However, the densification strain of the material could not be measured beyond a strain of 0.35, since the transverse area of the specimens was larger than the initial cross section area and irregular in shape. It is estimated that the densification strain should be in the range of 0.5–0.6 [29].

Table 4 shows the mechanical properties obtained from the compression tests (Figure 2) and the specimen dry densities after 28 days of curing. The results are presented as the mean ± standard deviation of at least three repetitions. In regard to the mixture densities, it can be seen that the highest density was 734 kg/m^3^, which is less than 5% of the target density. The PFC exhibited a compressive strength of 1.42 MPa. For the henequen fiber reinforced mixtures (Table 3), an increase in compressive strength with the increase in fiber content was observed. The highest compressive strength was obtained with the highest fiber volume fractions, i.e., increases of 22.5% and 25% were observed for the H1.5FC and HT1.5FC, respectively, when compared to the PFC. For the PP reinforced mixtures, an increase in strength of up to 51% was observed when compared to the PFC. It is noted that the compressive elastic strain was not accurately measured due to the lack of strain gauges. For a foamed concrete with a density of 700 kg/m^3^, the elastic modulus should be in the range of 1–1.4 GPa [42].

### 3.2. Uniaxial Tensile Test

**Figure 5:**
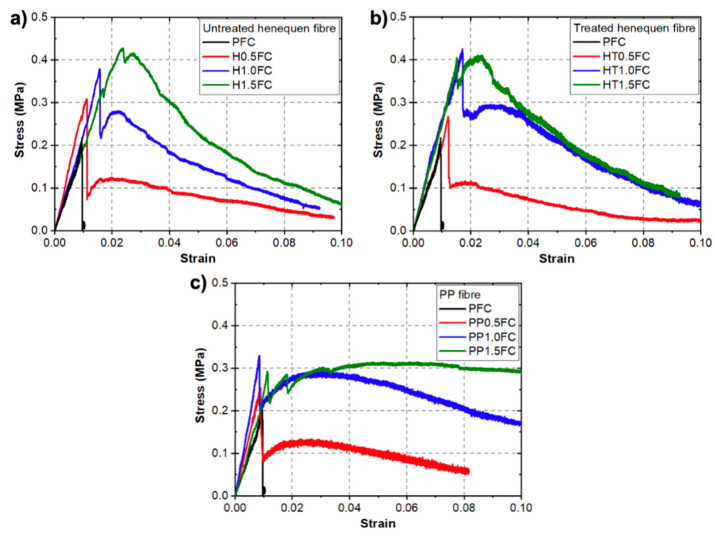
Typical uniaxial tensile stress–strain curves for reinforced foamed concrete mixtures with different fiber-reinforcements and volume fractions. A stress–strain curve of the PFC is included in each figure for comparison purposes: (**a**) untreated henequen fiber; (**b**) treated henequen fiber and (**c**) PP fiber.

Figure 5 shows typical stress–strain curves for the FRFCs from the tension tests. A stress–strain curve of the PFC is included in each figure for comparison purposes. It can be seen that for PFC, only an initial linear regime was observed until the peak strength was reached (0.22 MPa), which was followed by a sudden failure of the specimen (Figure 3b). For the FRFCs, the fibers prevented a sudden failure after the peak strength was reached. In general, an improvement in tensile strength was observed for the FRFCs when compared to the PFC. It can be seen in Figure 5 that FRFC curves exhibited the typical behavior of fiber reinforced cement-based materials for fiber volume fractions lower than 2% and fiber lengths of <25 mm [43], i.e., at the beginning of the test, a linear-elastic behavior was observed at low strains, followed by a sudden reduction in the stress corresponding to the first crack (failure of the matrix) and the onset of the plastic region. In the plastic region, the material can withstand a percentage of the maximum strength as the displacement increases, which is mainly attributed to frictional slip and pullout behavior [43]. Furthermore, it is noticed that the area under the curve for the PP fiber-reinforced mixtures increased with increasing fiber content. In the case of the FRFC reinforced with henequen fibers, similar increments were noticed; however, they were higher for the FRFC reinforced with the treated henequen fibers. This is an indication that the henequen fiber treatment is contributing to the increase of the material toughness, which is discussed in more detail in Section 3.4. It is noted that there is a small difference in the tensile behavior between 1% and 1.5% fiber contents, which is attributed to the fact that there was a reduction in the workability of the mixtures with 1.5% volume fraction. This may have resulted in poor fiber dispersion leading to no improvement or a reduction of the mechanical properties.

Table 5 shows the tensile properties obtained from the tension tests (Figure 5). The results are presented as the mean ± standard deviation of at least three repetitions. For the henequen fiber reinforced mixtures, the highest peak strength was observed for the mixtures with the treated fibers HT1.0FC and HT1.5FC (98% increase when compared to PFC). It is noted that the tensile peak strength was higher for the H0.5FC when compared to the HT0.5FC; however, when the strength was normalized by the density of the mixture (see Section 3.4), it can be seen that for a fiber volume fraction of 0.5%, the treatment had no influence on the tensile strength. For the PP fiber reinforced mixtures, the highest tensile strength was achieved with the volume fraction of 1% (52% increase when compared to PFC).

**Figure 6:**
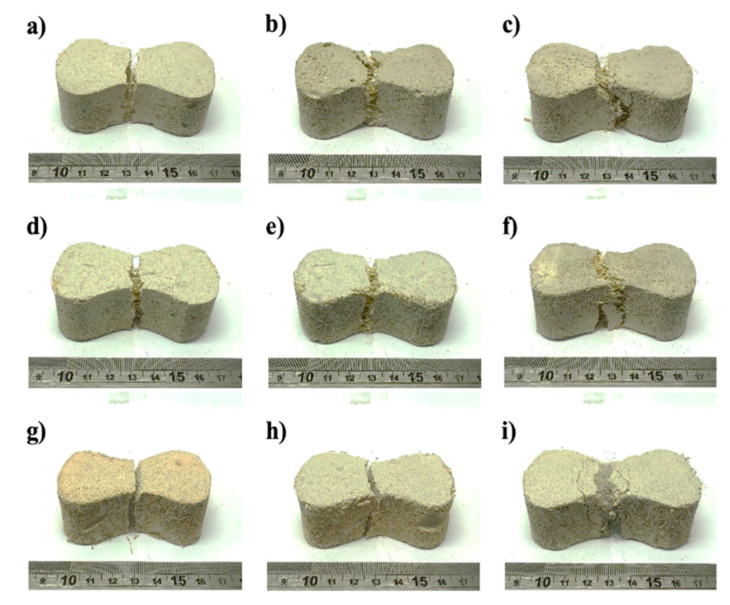
Representative foamed concrete specimens after the tensile test, reinforced with untreated henequen fiber: (**a**) H0.5FC; (**b**) H1.0FC and (**c**) H1.5FC; with treated henequen fiber: (**d**) HT0.5FC; (**e**) HT1.0FC and (**f**) HT1.5FC and with PP fiber: (**g**) PP0.5FC; (**h**) PP1.0FC and (**i**) PP1.5FC.

Figure 6 shows representative foamed concrete specimens after tensile testing, in which, the macroscopic cracking behavior was analyzed. It can be seen that for all mixtures with 0.5% fiber volume fraction (Figure 6a,d,g), there was only one straight crack at the waist line of the briquette; however, for 1.0% volume fraction (Figure 6b,e,h) the single crack followed a tortuous path, which caused an increase in the toughness (Figure 5). For the specimens with 1.5% volume fraction, it can be observed that there was not only a main crack that followed a tortuous path, but also several secondary cracks, which in turn resulted in a large increase in toughness. For PP1.5FC, small cracks were also observed (Figure 6i). The cracking behavior could have been affected by the fiber–matrix bond and the effect of the fiber type and content on the air-void structure. A study of the cracking behavior of FRFC that takes into account these factors is recommended, which was beyond the scope of this work.

### 3.3. Microscopic Characterization of Air-Voids

**Figure 7:**
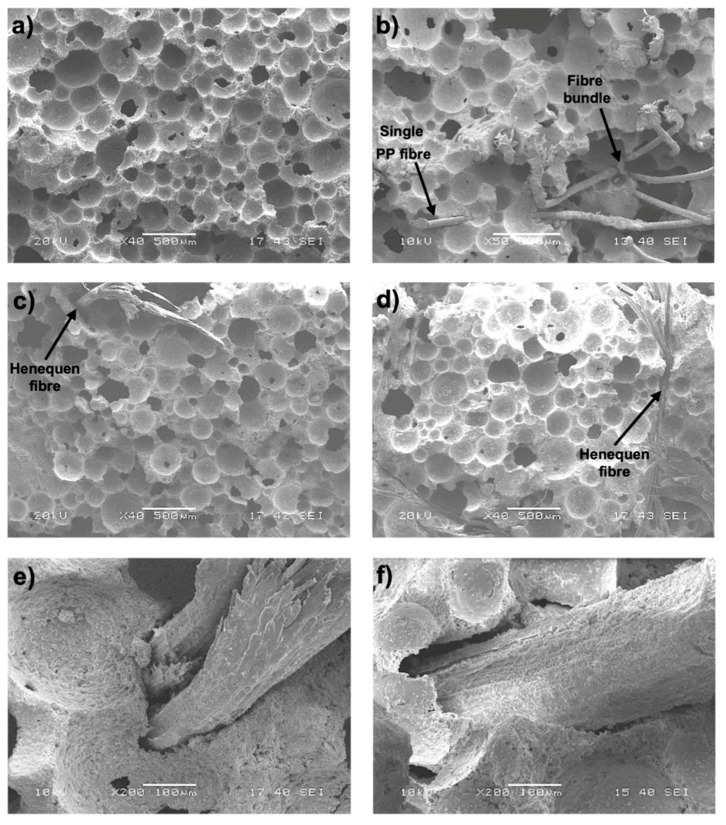
SEM images of fiber-reinforced foamed concrete (FRFC): (**a**) PFC; (**b**) PP1.5FC; (**c**) H1.5FC; (**d**) HT1.5FC; (**e**) single untreated henequen fiber in the FRFC and (**f**) single treated henequen fiber in the FRFC.

SEM images of the microstructure of the PFC and FRFCs with a 1.5% fiber volume fraction are shown in Figure 7. It can be seen that for all foamed concrete mixtures, a similar air-void structure and air-void size were observed notwithstanding the high content of fiber. This is explained by the fact that the air-void size depends mainly on the target dry density, i.e., the amount of foam added to the mix. Table 6 shows the average air-void diameters that were obtained for each mixture from the statistical analysis of the SEM images. It can be observed that there was not a significant variation in the equivalent diameter of the air-voids among the different mixtures. The smallest equivalent diameter was observed for the PP1.5FC (214 µm) while the largest equivalent diameter was observed for the H1.5FC (235 µm). Figure 7a shows the microstructure of the PFC while Figure 7b shows the microstructure of the PP1.5FC. Figure 7b suggests that single PP fibers do not affect the size of the air-voids around them, i.e., the microstructure of PP1.5FC was similar to that of the PFC (Figure 7a); however, it can also be seen in Figure 7b that for fiber bundles, the surrounding air-voids tended to be larger, which highlighted the importance of adequate fiber dispersion in the matrix. Figure 7c,d shows the microstructure of H1.5FC and HT1.5FC, respectively. These figures show that the microstructure of the mixtures with henequen fibers was similar to that of the PFC; however, Figure 7e,f, which shows a close-up of the air-voids around single untreated and treated henequen fibers, respectively, suggests that the air-voids around the henequen fibers were interconnected to form larger air-voids, which may be explained by the larger diameter of the henequen fiber, when compared to the PP fiber. This effect is less pronounced for the treated fiber; however, further microscopy studies should be performed to confirm this observation. The PP fiber diameter was 70 µm while the henequen fiber diameter was 170 µm. The former was smaller than the smallest air-void diameter (223.7 ± 77.4 µm) while the latter was in the middle between the smallest and the average air-void diameters. This suggests that there may be a considerable number of air-voids connected by the henequen fibers. This also suggests that when fibers with a smaller diameter were used (Figure 7b), the performance of FRFC might increase; however, to verify this, a comprehensive microstructural analysis, including pullout testing, nanoindentation and further microscopic studies, should be performed to take into account other important factors such as fiber–matrix bonding and mechanical properties of the fiber, which was beyond the scope of this work.

**Figure 8:**
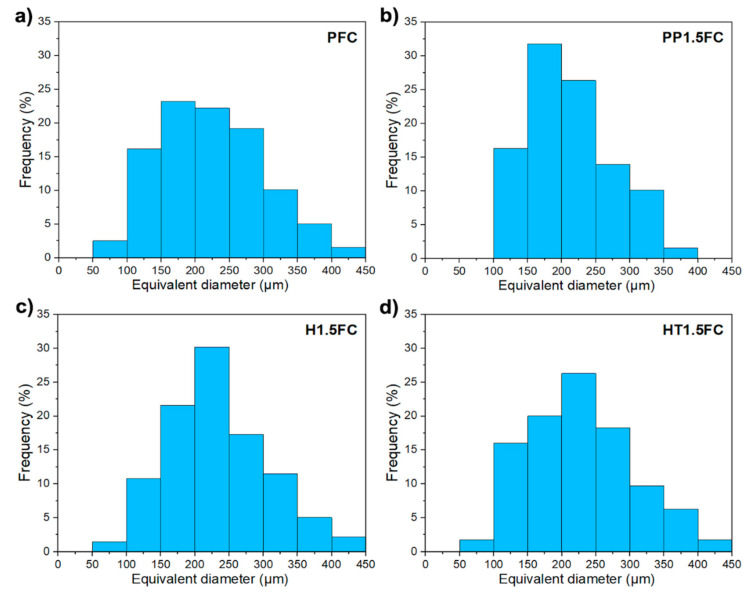
Frequency histograms of the air-void diameters of foamed concrete mixtures: (**a**) PFC; (**b**) PP1.5FC; (**c**) H1.5FC and (**d**) HT1.5FC.

Figure 8 shows the frequency histograms of the air-void diameters values for the PFC and FRFCs with a 1.5% fiber volume fraction. It can be seen that the histograms were left-skewed and most of the data were concentrated between the 150 and 250 µm classes. Air-voids with diameter values above 400 µm can be observed in the histograms, which could be the result of interconnected air-voids. These results showed that inclusion of fibers in the foamed concrete mixtures, either henequen or polypropylene, did not significantly modify the cellular structure of the foamed concrete since similar air-void sizes and their distributions were observed in all mixtures.

### 3.4. Discussion

**Figure 9:**
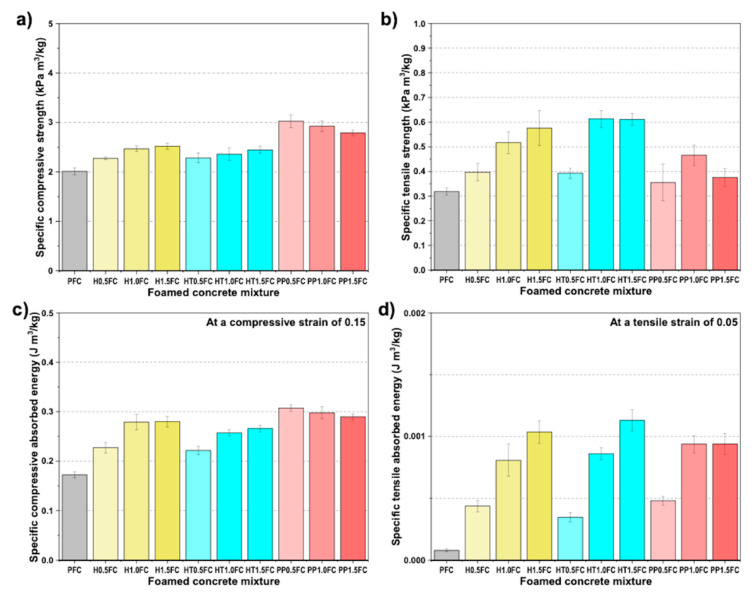
Normalized mechanical properties of foamed concrete mixtures: (**a**) specific compressive strength (SCS); (**b**) specific tensile strength (STS); (**c**) specific compressive absorbed energy (SCAE) and (**d**) specific tensile absorbed energy (STAE).

Figure 9a shows the specific compressive strength (SCS) for all mixtures, which was obtained through the normalization of the strength by the corresponding density of the mixture (Table 4). The normalized strength was used to take into account any changes in the mechanical properties due to the variations in density among the different mixtures. From Figure 9a, it can be seen that the fiber-reinforcement enhanced the SCS in all mixtures when compared to the PFC, which is attributed to the enhanced specimen integrity by the fibers [6]. The fiber-reinforcement enables a distributed growth of microcracks in the specimen prior to the development of macrocracks [28]. It can also be seen in the Figure 9a that the SCS increased with the increase in henequen fiber content and decreased with the increase in PP fiber content. It is believed that for PP fiber volume fractions >1.0%, there was not good fiber dispersion, which in turn, promoted fiber bundles and large air-voids. Figure 9b shows the specific tensile strength (STS) for all mixtures. It can be seen that the fiber-reinforcement enhanced the STS in all mixtures when compared to the PFC. For the henequen fiber-reinforced specimens, the STS increased with the increase in fiber content in all cases. For the PP fiber-reinforced specimens, the STS increased with the increase in fiber content from 0.5% to 1% and decreased when the fiber content increased from 1% to 1.5%. It is noted that the enhancement of the STS in the henequen fiber-reinforced specimens was higher than that of the PP fiber-reinforced specimens. This was attributed to the higher elastic modulus of the henequen fiber when compared to the PP fiber. It is also noted that in all cases the fiber inclusion did not affect the overall air-void size and its distribution (see Section 3.3), which is very important since they affect the mechanical properties of foamed concrete [44]. It has been reported that increasing the fiber content in a cement-based matrix could affect the air-void structure by producing a coarser pore structure and increasing the number of pores [8,45], which in turn could affect the mechanical properties of the fiber-reinforced material. The use of low fiber volume fractions and adequate fiber dispersion in the cementitious matrix will normally result in good workability and an increase of the mechanical properties. SEM images suggest that air-voids were locally larger around henequen fibers, which was attributed to the larger diameter of the henequen fiber when compared to that of the PP fiber; however, further studies should be performed to confirm this observation and to assess how this effect affects the overall performance of the FRCFs.

Figure 9b also shows that the alkaline treatment of the henequen fibers increased the tensile strength of the mixtures with 1% and 1.5% fiber volume fraction when compared to the corresponding mixtures with untreated fibers. The better performance exhibited by the HT1.0FC and HT1.5FC could be attributed to the fact that the alkaline treatment partially eliminates the hemicellulose and lignin present on the surface of the fibers [38], producing a fiber with a rougher surface, and consequently, a better mechanical interlocking is created between the fibers and the matrix [38]. The rougher surface of the treated fiber may also produce some frictional resistance to extraction after debonding, when compared to the untreated fiber. The use of alkaline solutions on natural fibers removes most of the surface non-cellulosic materials and impurities, increasing the roughness of the fiber surface and the fiber–matrix bond and producing a higher frictional pullout [46]. It is also possible that the alkaline treatment improved the tensile strength and Young’s modulus of the treated fibers by increasing their cellulose crystallinity [46]; however, microstructural analysis and further mechanical testing are needed to understand the interaction of the henequen fiber with the cement-based matrix. It is emphasized that in terms of durability, treatment of the fibers is crucial when used as reinforcement of cement-based materials. The workability of the foamed concrete mixtures decreased with the inclusion of the fibers when compared to the PFC. However, the workability of the mixtures decreased substantially when a fiber volume fraction of 1.5% was used. For this reason, a maximum fiber volume fraction of 1% is recommended for the cases studied here, in terms of a trade-off between mechanical properties enhancement and loss of workability.

Figure 9c,d shows the specific compressive absorbed energy (SCAE) at a compressive strain of 0.15 and the specific tensile absorbed energy (STAE) at a tensile strain of 0.05, respectively. SCAE and STAE were obtained through the normalization of the absorbed energies by the corresponding density of the mixture (Table 4). The absorbed energies were estimated as the area under the compression and tension force–displacement curves up to a strain of 0.15 and 0.05, respectively. These results clearly show the energy absorption enhancement of the FRFCs when compared to the PFC. The enhancement is more evident for the STAE, in which, the fibers play a crucial role in the increase in ductility of the FRFC specimens. The toughness improvement in the tensile behavior of the FRFCs occurs through the absorption of energy related to the debonding and extraction of the fibers bridging the cracks [43]. This changes the failure mode of the foamed concrete from brittle to ductile, which is very important in order to avoid catastrophic failure when the material is in service. This improvement in STAE was higher for the FRFC reinforced with the treated henequen fiber (STAE increased 6.6% and 9.1% for HT1.0FC and HT1.5FC when compared to H1.0FC and H1.5FC, respectively), which suggests that the treatment is contributing to the increase of the material toughness by increasing the roughness of the fiber surface, which in turn produced an improved fiber–matrix bond and higher frictional pullout. However, as discussed earlier, a comprehensive microstructural analysis is needed to understand the interaction of the treated henequen fiber with the cement-based matrix and the effect of the treated fiber content on the mechanical properties of FRFC.

The results presented here have shown that henequen fiber can be used to obtain low density foamed concrete with improved mechanical properties, which could promote a lower environmental footprint and sustainability in the construction industry. Although this lightweight FRFC has low strength when compared to ordinary concrete or other cellular concretes with higher densities, this material could be used for various applications, due to its low density and physical properties, which include seismic [30] and impact energy absorption applications [32]. This material could also be used for the manufacture of precast partition walls and lightweight blocks, or as the core material for sandwich composites [6]. One of the main concerns in the development of natural fiber reinforced mortars is the durability of the fibers in the alkaline environment of cement. Several studies have shown that the durability of these materials can be improved by treating the fibers with chemical or thermal treatments [22,24]. The fire resistance of natural fiber reinforced cement-based materials should also be investigated, considering that research addressing this issue remains scarce [47]. Our results warrant further research of foamed concrete reinforced with henequen fibers. These studies should include strategies to improve durability and further mechanical testing to fully understand the toughening mechanisms governing the macroscopic mechanical properties of foamed concrete reinforced with natural fibers.

## 4. Conclusions

Uniaxial compression and tension tests were performed on plain foamed concrete (PFC) and fiber-reinforced foamed concrete (FRFC) with a target dry density of 700 kg/m^3^. The foamed concrete was reinforced with henequen natural fibers and PP fibers at volume fractions of 0.5%, 1% and 1.5%. The following conclusions can be drawn from this study:

- For all FRFC mixtures, the inclusion of the fibers enhanced the compressive strength and plastic behavior, which was attributed to the increase of specimen integrity by the fibers;
- For all FRFC mixtures, after the peak compressive strength, there was no considerable loss in strength and a plateau-like regime was observed;
- The tension tests showed that fiber-reinforcement significantly increased the tensile strength of the FRFCs and prevented a sudden failure of the specimens, which was in contrast to the brittle behavior of the PFC;
- The tensile strength was higher when treated henequen fibers at 1% and 1.5% volume fractions were used, which was attributed to a better fiber–matrix interaction due to the alkaline treatment of the fibers;
- The workability of the mixtures decreased substantially for a fiber volume fraction of 1.5%;
- The microscopic characterization showed that the inclusion of fibers did not significantly modify the cellular structure of the foamed concrete;
- A much higher energy absorption was observed for FRFCs when compared to the PFC, which is attributed to the increased toughness and ductility produced by the fibers;
- The specific tensile absorbed energy at a tensile strain of 0.05 was higher when treated henequen fibers at a 1.5% volume fraction were used, which suggested that the treatment contributed to the increase of the material toughness;
- The mechanical results indicated that FRFC reinforced with henequen fiber has the potential to be used as a sustainable lightweight construction material, and to be used as the core material for sandwich composites.

## Figures and Tables

**Table 1:** Constituent proportions of foamed concrete for 1 m^3^.

Constituent	Content (kg)
Cement	342
Limestone aggregate	171
Water	240
Foam	29

**Table 2:** Mechanical properties of the henequen [38] and PP [37] fibers.

Fiber	Density (kg/m^3^)	Tensile Strength (MPa)	Elastic Modulus (GPa)	Diameter (µm)	Length (mm)
Henequen	1400	500	13.2	170	19
PP	900	552	3.8	70	19

**Table 3:** Foamed concrete mixtures for the mechanical characterization.

Mixture	Type of Fiber	Volume Fraction (%)
PFC	-	-
H0.5FC	Henequen	0.5
H1.0FC	Henequen	1
H1.5FC	Henequen	1.5
HT0.5FC	Treated henequen	0.5
HT1.0FC	Treated henequen	1
HT1.5FC	Treated henequen	1.5
PP0.5FC	Polypropylene	0.5
PP1.0FC	Polypropylene	1
PP1.5FC	Polypropylene	1.5

**Table 4:** Uniaxial compression test results.

Mixture	Elastic Modulus (MPa)	Compressive Strength (MPa)	Yield Strain (mm/mm)	Density (kg/m^3^)
PFC	230 ± 9	1.42 ± 0.05	0.007 ± 0.001	706 ± 8
H0.5FC	218 ± 25	1.62 ± 0.02	0.008 ± 0.002	713 ± 5
H1.0FC	277 ± 21	1.68 ± 0.04	0.007 ± 0.001	681 ± 1
H1.5FC	196 ± 31	1.74 ± 0.04	0.010 ± 0.002	691 ± 4
HT0.5FC	224 ± 21	1.58 ± 0.07	0.008 ± 0.001	692 ± 3
HT1.0FC	219 ± 23	1.72 ± 0.09	0.012 ± 0.001	729 ± 3
HT1.5FC	269 ± 27	1.78 ± 0.05	0.009 ± 0.002	728 ± 7
PP0.5FC	254 ± 30	2.06 ± 0.09	0.008 ± 0.001	681 ± 13
PP1.0FC	278 ± 19	2.14 ± 0.08	0.010 ± 0.002	734 ± 1
PP1.5FC	275 ± 25	2.04 ± 0.04	0.010 ± 0.001	732 ± 1

**Table 5:** Uniaxial tensile test results.

Mixture	Elastic Modulus (MPa)	Tensile Strength (MPa)	Yield Strain (mm/mm)
PFC	19.81 ± 2.5	0.225 ± 0.01	0.010 ± 0.001
H0.5FC	27.96 ± 3.53	0.283 ± 0.025	0.011 ± 0.001
H1.0FC	20.69 ± 2.42	0.352 ± 0.03	0.019 ± 0.003
H1.5FC	23.31 ± 4.09	0.398 ± 0.049	0.013 ± 0.002
HT0.5FC	20 ± 1.76	0.272 ± 0.014	0.013 ± 0.002
HT1.0FC	25.95 ± 3.01	0.447 ± 0.025	0.016 ± 0.003
HT1.5FC	23.51 ± 1.90	0.445 ± 0.017	0.016 ± 0.002
PP0.5FC	22.81 ± 2.4	0.242 ± 0.05	0.011 ± 0.001
PP1.0FC	30.35 ± 4.19	0.341 ± 0.03	0.010 ± 0.003
PP1.5FC	24.85 ± 3.64	0.275 ± 0.03	0.012 ± 0.002

**Table 6:** Average equivalent air-void diameter of the mixtures.

Mixture	Equivalent Diameter (µm)	Porosity of Air-Voids (%)
PFC	223.69 ± 77.37	59.02
H0.5FC	229.30 ± 73.66	58.62
H1.0FC	226.12 ± 71.51	60.48
H1.5FC	235.45 ± 73.64	59.90
HT0.5FC	221.30 ± 67.44	59.84
HT1.0FC	226.47 ± 66.90	57.69
HT1.5FC	228.99 ± 78.61	57.75
PP0.5FC	224.37 ± 68.84	60.48
PP1.0FC	216.68 ± 61.99	57.40
PP1.5FC	214.03 ± 60.59	57.52

## References

[1] Ramamurthy K., Kunhanandan Nambiar E.K., Indu Siva Ranjani G. (2009). A classification of studies on properties of foam concrete. Cem. Concr. Compos..

[2] Mugahed Amran Y.H., Farzadnia N., Abang Ali A.A. (2015). Properties and applications of foamed concrete; a review. Constr. Build. Mater..

[3] Song P.S., Hwang S., Sheu B.C. (2005). Strength properties of nylon- and polypropylene-fiber-reinforced concretes. Cem. Concr. Res..

[4] Falliano D., De Domenico D., Ricciardi G., Gugliandolo E. (2019). Compressive and flexural strength of fiber-reinforced foamed concrete: Effect of fiber content, curing conditions and dry density. Constr. Build. Mater..

[5] Arisoy B., Wu H.-C. (2008). Material characteristics of high performance lightweight concrete reinforced with PVA. Constr. Build. Mater..

[6] Flores-Johnson E.A., Li Q.M. (2012). Structural behaviour of composite sandwich panels with plain and fibre-reinforced foamed concrete cores and corrugated steel faces. Compos. Struct..

[7] Jones M.R., McCarthy A. (2005). Preliminary views on the potential of foamed concrete as a structural material. Mag. Concr. Res..

[8] Othuman Mydin M.A., Soleimanzadeh S. (2012). Effect of polypropylene fiber content on flexural strength of lightweight foamed concrete at ambient and elevated temperatures. Adv. Appl. Sci. Res..

[9] Mugahed Amran Y.H., Alyousef R., Alabduljabbar H., Khudhair M.H.R., Hejazi F., Alaskar A., Alrshoudi F., Siddika A. (2020). Performance properties of structural fibred-foamed concrete. Results Eng..

[10] Guerini V., Conforti A., Plizzari G., Kawashima S. (2018). Influence of Steel and Macro-Synthetic Fibers on Concrete Properties. Fibers.

[11] Wu Y., Song W., Zhao W., Tan X. (2018). An Experimental Study on Dynamic Mechanical Properties of Fiber-Reinforced Concrete under Different Strain Rates. Appl. Sci..

[12] Hoyos C.G., Zuluaga R., Gañán P., Pique T.M., Vazquez A. (2019). Cellulose nanofibrils extracted from fique fibers as bio-based cement additive. J. Cleaner Prod..

[13] Chiacchiarelli L.M., Cerrutti P., Flores-Johnson E.A. (2020). Compressive behavior of rigid polyurethane foams nanostructured with bacterial nanocellulose at low and intermediate strain rates. J. Appl. Polym. Sci..

[14] Frydrych M., Hýsek Š., Fridrichová L., Le Van S., Herclík M., Pechočiaková M., Le Chi H., Louda P. (2020). Impact of Flax and Basalt Fibre Reinforcement on Selected Properties of Geopolymer Composites. Sustainability.

[15] Kandemir A., Pozegic T.R., Hamerton I., Eichhorn S.J., Longana M.L. (2020). Characterisation of Natural Fibres for Sustainable Discontinuous Fibre Composite Materials. Materials.

[16] Ramakrishna G., Sundararajan T. (2005). Impact strength of a few natural fibre reinforced cement mortar slabs: A comparative study. Cem. Concr. Compos..

[17] Ahmad W., Farooq S.H., Usman M., Khan M., Ahmad A., Aslam F., Yousef R.A., Abduljabbar H.A., Sufian M. (2020). Effect of Coconut Fiber Length and Content on Properties of High Strength Concrete. Materials.

[18] Okeola A.A., Abuodha S.O., Mwero J. (2018). Experimental Investigation of the Physical and Mechanical Properties of Sisal Fiber-Reinforced Concrete. Fibers.

[19] Tolêdo Filho R.D., Scrivener K., England G.L., Ghavami K. (2000). Durability of alkali-sensitive sisal and coconut fibres in cement mortar composites. Cem. Concr. Compos..

[20] Wei J., Meyer C. (2015). Degradation mechanisms of natural fiber in the matrix of cement composites. Cem. Concr. Res..

[21] Fidelis M.E.A., Toledo Filho R.D., Silva F.d.A., Mechtcherine V., Butler M., Hempel S. (2016). The effect of accelerated aging on the interface of jute textile reinforced concrete. Cem. Concr. Compos..

[22] De Klerk M.D., Kayondo M., Moelich G.M., de Villiers W.I., Combrinck R., Boshoff W.P. (2020). Durability of chemically modified sisal fibre in cement-based composites. Constr. Build. Mater..

[23] Claramunt J., Ardanuy M., García-Hortal J.A., Tolêdo Filho R.D. (2011). The hornification of vegetable fibers to improve the durability of cement mortar composites. Cem. Concr. Compos..

[24] Wei J., Meyer C. (2014). Improving degradation resistance of sisal fiber in concrete through fiber surface treatment. Appl. Surf. Sci..

[25] Mahzabin M.S., Hock L.J., Hossain M.S., Kang L.S. (2018). The influence of addition of treated kenaf fibre in the production and properties of fibre reinforced foamed composite. Constr. Build. Mater..

[26] Othuman Mydin M.A., Rozlan N.A., Ganesan S. (2015). Experimental study on the mechanical properties of coconut fibre reinforced lightweight foamed concrete. J. Mater. Environ. Sci..

[27] Liu Y., Wang Z., Fan Z., Gu J. (2020). Study on properties of sisal fiber modified foamed concrete. IOP Conf. Ser. Mater. Sci. Eng..

[28] Amarnath Y., Ramachandrudu C. Properties of Foamed Concrete with Sisal Fibre. Proceedings of the 9th International Concrete Conference 2016: Environment, Efficiency and Economic Challenges for Concrete.

[29] Flores-Johnson E.A., Yan Y.Z., Carrillo J.G., González-Chi P.I., Herrera-Franco P.J., Li Q.M. (2018). Mechanical Characterization of Foamed Concrete Reinforced with Natural Fibre. Mater. Res. Proc..

[30] Flores-Johnson E.A., Company-Rodríguez B.A., Koh-Dzul J.F., Carrillo J.G. (2020). Shaking Table Test of U-Shaped Walls Made of Fiber-Reinforced Foamed Concrete. Materials.

[31] Sayadi A.A., Tapia J.V., Neitzert T.R., Clifton G.C. (2016). Effects of expanded polystyrene (EPS) particles on fire resistance, thermal conductivity and compressive strength of foamed concrete. Constr. Build. Mater..

[32] Feng S., Zhou Y., Wang Y., Lei M. (2020). Experimental research on the dynamic mechanical properties and damage characteristics of lightweight foamed concrete under impact loading. Int. J. Impact Eng..

[33] Dewey L.H. (1931). Sisal and Henequen, Plants Yielding Fiber for Binder Twine. https://naldc.nal.usda.gov/catalog/ORC00000571.

[34] CEMEX (2020). Cemento CPC 30R Extra Data Sheet, Cemex, Monterrey, Mexico. https://www.cemexmexico.com/documents/27057941/45887874/ficha-digital-CPC-30R-Extra.pdf.

[35] Vázquez-Rodríguez F.J., Elizondo-Villareal N., Verástegui L.H., Arato Tovar A.M., López-Perales J.F., Contreras de León J.E., Gómez-Rodríguez C., Fernández-González D., Verdeja L.F., García-Quiñonez L.V. (2020). Effect of Mineral Aggregates and Chemical Admixtures as Internal Curing Agents on the Mechanical Properties and Durability of High-Performance Concrete. Materials.

[36] Solís-Carcaño R., Moreno E.I. (2006). Análisis de la porosidad del concreto con agregado calizo. Rev. Fac. Ing. UCV.

[37] Dificonsa (2018). Fibercon Microfibra (In Spanish), Technical Data Sheet, Dificonsa. https://dificonsa.com/HDE/FT-FiberconMicrofibra.pdf.

[38] Valadez-Gonzalez A., Cervantes-Uc J., Olayo R., Herrera-Franco P. (1999). Effect of fiber surface treatment on the fiber–matrix bond strength of natural fiber reinforced composites. Compos. Part B.

[39] Kearsley E., Visagie M. (2002). Properties of foamed concrete as influenced by air-void parameters. Concr. Beton.

[40] Wei S., Yiqiang C., Yunsheng Z., Jones M.R. (2013). Characterization and simulation of microstructure and thermal properties of foamed concrete. Constr. Build. Mater..

[41] Flores-Johnson E.A., Li Q.M., Mines R.A.W. (2008). Degradation of elastic modulus of progressively crushable foams in uniaxial compression. J. Cell. Plast..

[42] Kozłowski M., Kadela M. (2018). Mechanical Characterization of Lightweight Foamed Concrete. Adv. Mater. Sci. Eng..

[43] Tolêdo Filho R.D., Joseph K., Ghavami K., England G.L. (1999). The use of sisal fibre as reinforcement in cement based composites. Rev. Bras. Eng. Agric. Ambient..

[44] Nguyen T.T., Bui H.H., Ngo T.D., Nguyen G.D., Kreher M.U., Darve F. (2019). A micromechanical investigation for the effects of pore size and its distribution on geopolymer foam concrete under uniaxial compression. Eng. Fract. Mech..

[45] Ramezanianpour A.A., Esmaeili M., Ghahari S.A., Najafi M.H. (2013). Laboratory study on the effect of polypropylene fiber on durability, and physical and mechanical characteristic of concrete for application in sleepers. Constr. Build. Mater..

[46] Ferreira S.R., Silva F.d.A., Lima P.R.L., Toledo Filho R.D. (2015). Effect of fiber treatments on the sisal fiber properties and fiber–matrix bond in cement based systems. Constr. Build. Mater..

[47] Netinger Grubeša I., Marković B., Gojević A., Brdarić J. (2018). Effect of hemp fibers on fire resistance of concrete. Constr. Build. Mater..

